# Joining Caffeic Acid and Hydrothermal Treatment to Produce Environmentally Benign Highly Reduced Graphene Oxide

**DOI:** 10.3390/nano11030732

**Published:** 2021-03-15

**Authors:** Ana Barra, Oana Lazăr, Aida Pantazi, María J. Hortigüela, Gonzalo Otero-Irurueta, Marius Enăchescu, Eduardo Ruiz-Hitzky, Cláudia Nunes, Paula Ferreira

**Affiliations:** 1 Department of Materials and Ceramic Engineering, CICECO—Aveiro Institute of Materials, University of Aveiro, 3810-193 Aveiro, Portugal; abarra@ua.pt; 2 Department of Chemistry, CICECO—Aveiro Institute of Materials, University of Aveiro, 3810-193 Aveiro, Portugal; 3 Materials Science Institute of Madrid, CSIC, c/Sor Juana Inés de la Cruz 3, 28049 Madrid, Spain; eduardo@icmm.csic.es; 4 Center for Surface Science and Nanotechnology, University Politehnica of Bucharest, 060042 Bucharest, Romania; oana.lazar@cssnt-upb.ro (O.L.); aida.pantazi@cssnt-upb.ro (A.P.); marius.enachescu@cssnt-upb.ro (M.E.); 5 S.C. NANOPRO START M.C. S.R.L., 110310 Pitești, Romania; 6 Centre for Mechanical Technology & Automation, University of Aveiro, 3810-193 Aveiro, Portugal; mhortiguela@ua.pt (M.J.H.); otero.gonzalo@ua.pt (G.O.-I.); 7 Academy of Romanian Scientists, 50085 Bucharest, Romania

**Keywords:** reduced graphene oxide, caffeic acid, hydrothermal reduction, carbon spheres, sustainable

## Abstract

Reduced graphene oxide (rGO) is a promising graphene-based material, with transversal applicability to a wide range of technological fields. Nevertheless, the common use of efficient—but hazardous to environment and toxic—reducing agents prevents its application in biological and other fields. Consequently, the development of green reducing strategies is a requirement to overcome this issue. Herein, a green, simple, and cost-effective one-step reduction methodology is presented. Graphene oxide (GO) was hydrothermally reduced in the presence of caffeic acid (CA), a natural occurring phenolic compound. The improvement of the hydrothermal reduction through the presence of CA is confirmed by XRD, Raman, XPS and TGA analysis. Moreover, CA polymerizes under hydrothermal conditions with the formation of spherical and non-spherical carbon particles, which can be useful for further rGO functionalization. FTIR and XPS confirm the oxygen removal in the reduced samples. The high-resolution scanning transmission electron microscopy (HRSTEM) images also support the reduction, showing rGO samples with an ordered graphitic layered structure. The promising rGO synthesized by this eco-friendly methodology can be explored for many applications.

## 1. Introduction

Graphene is a one-atom-thick, two-dimensional (2D) carbon material composed of hybridized sp^2^ carbon atoms organized in a hexagonal lattice, with exceptional thermal [1], optical [2], mechanical [3] and electrical properties [4]. The combination of all these properties in only one material triggered the research around graphene derivatives, enlarging its application to distinct technological fields such as energy production [5], environmental remediation [6] or medicine [7].

The fabrication of graphene materials is categorized into bottom–up and top–down approaches. In the bottom–up methods, atoms and molecules are building blocks used to form a material with higher dimensionality, such as the use of 0D hydrocarbon molecules to synthesize 2D graphene sheets. In opposition, the top–down methods are based on the deconstruction of a bulk material to form another material with lower dimensionality, for example, the reduction of graphene oxide [8]. The reduction of graphene oxide is a technique in which graphite is oxidized using strong acids to form graphene oxide (GO) [9], which is then reduced to remove the oxygen groups and form reduced graphene oxide (rGO). The reduction step is usually achieved by thermal [10] or chemical treatments [11], but can be enhanced by a combination of both methods, allowing the use of milder reductants and lower temperatures [12,13].

Hydrazine is the most popular reducing agent, due to its efficient reducing ability [14]. Nevertheless, this compound is highly toxic, and its use prevents the further application of rGO in sensitive biological areas, namely the biomedical one. The research of eco-friendly alternatives to reduce GO have been investigated, avoiding toxic reducing agents and high energy consumption methodologies.

Nature provides a wide range of non-toxic reducing agents. Plant extracts containing phytomolecules [15], vitamins [16] or phenolic compounds [17] are described in the literature as being able to simultaneously reduce and stabilize GO. Caffeic acid (3,4-dihydroxycinnamic acid) is a natural phenolic acid widely distributed in plants [18,19]. The antioxidant activity of caffeic acid (CA) is related to its structure, namely the presence of two hydroxyl groups at the positions 3 and 4 of the aromatic ring and a double bond conjugated in the side chain [20,21]. The non-toxicity and high antioxidant activity of CA turns it attractive to be used as GO reductant. Bo and co-workers [22], reduced GO with CA and water as solvent at 95 °C for 24 h, using a GO:CA ratio of 1:50. The low oxygen content present in rGO pointed CA to be among the best green reducing agents.

The hydrothermal reduction of graphene oxide is a thermal treatment that takes place inside a closed vessel in an aqueous medium heated at subcritical temperatures (120–200 °C) under self-generated pressure. The superheated water promotes the cleavage of the oxygen functionalities and restores the graphitic structure [23,24]. Thus, the hydrothermal reduction method is considered a simple, scalable, and eco-friendly strategy. However, the hydrothermal treatment and the green chemical reduction are mild reduction strategies that partially remove the oxygen groups present in GO. Nonetheless, these chemical functionalities are useful to further disperse rGO or as functionalization sites [25]. The combination of the hydrothermal and chemical reduction provides the opportunity to simultaneously reduce and functionalize the rGO. Long et al. [26] described the simultaneous oxygen reduction of GO and nitrogen doping of rGO by the hydrothermal treatment of GO in the presence of hydrazine and ammonia solution. In addition, the hydrothermal treatment can also improve the reduction efficiency of the green chemical reductants. Glucose and ammonium hydroxide were used to reduce GO under hydrothermal conditions. The synthesized rGO contained less oxygen than a rGO sample reduced with hydrazine [27]. The hydrothermal treatment of GO in the presence of ascorbic acid at 160 °C during 4 h improved the known reducing ability of the ascorbic acid and provided rGO with an electrical conductivity of ~5 S m^−1^ [28].

In this work, the effect of coupling in one step the known high reducing capacity of CA to the hydrothermal conditions on the reduction of graphene oxide under environmental benign conditions was studied. Different CA and GO ratios-based materials were successfully prepared and investigated, and the optimal conditions to maximize the level of reduction were found. A detailed characterization of the morphology, structure, and thermal properties of GO and rGO samples is provided. This green, simple, and cost-effective reduction method enables the applicability of the rGO in biological, medical and/or food fields.

## 2. Materials and Methods

### 2.1. Chemicals

Graphite flakes (particle size ~150 µm), sulfuric acid (97%), phosphoric acid (≥85%), potassium permanganate (99,0%), hydrochloric acid (37%), hydrogen peroxide (30%) and caffeic acid (≥95%) were purchased from Sigma–Aldrich Co. (St Louis, MO, USA) and used as received.

### 2.2. Synthesis of GO

Graphene oxide was synthesized by the improved Hummers method [9]. Graphite flakes (1.5 g) were mixed with sulphuric (180 mL) and phosphoric acid (20 mL), followed by the slow addition of potassium permanganate (9 g) and the mixture was kept under stirring in an oil bath at 50 °C overnight. The mixture was transferred to an ice bath and ultrapure water (200 mL) at 4 °C was added followed by the addition of hydrogen peroxide (3 mL). The resultant mixture was centrifuged. The solid material was sequentially washed by centrifugation during 20 min at 6000 rpm, with ultrapure water (200 mL), 30% hydrochloric acid (200 mL) and ethanol (200 mL × 2). The final GO solution was dispersed in ultrapure water with a Sonoplus HD 3100 ultrasound probe (45 W, 1 h) (Bandelin, Berlim, Germany).

### 2.3. Reduction of GO

GO was hydrothermally reduced in the presence of different amounts of CA, used as a chemical reductant. 40 mL of a GO solution (7.5 mg/mL) and CA were placed inside a Teflon lined autoclave at 180 °C during 24 h. After the reduction, the autoclave was naturally cooled down to room temperature. The resultant black material was washed several times with distilled water, separated by filtration and dried in an oven at 60 °C overnight. The samples were denoted as GO:CA (X:Y), with “X” and “Y” being the mass ratio of GO and CA, respectively. Five different GO:CA mass ratios were tested: GO:CA (1:0); GO:CA (1:0.1); GO:CA (1:0.5); GO:CA (1:1) and GO:CA (0:1).

### 2.4. Characterization of rGO

X-ray diffraction (XRD) analyses were carried out on a SmartLab X-ray diffractometer (Rigaku Corp., Tokyo, Japan). The high-resolution XRD patterns were measured at 9 kW (45 kV and 200 mA) with Cu target Kα radiation (λ = 0.15406 nm) and recorded in the (4°–70°) 2*θ* range. The samples were analyzed in Bragg Brentano Geometry (continuous mode) with 0.01° step size and scanning speed of 3° (2*θ*)/min.

Raman spectroscopy investigations were carried out using a LabRam HR800 Confocal micro-Raman Spectrometer (Horiba Ltd., Kyoto, Japan), at room temperature. All the Raman spectra were generated using a 532 nm wavelength green excitation laser and by dispersing the sample emitted signal onto the Charge Coupled Device (CCD) detector using a 600 lines/mm grating.

X-ray photoelectron spectroscopy (XPS) was performed in an ultra high vacuum (UHV) system with a base pressure of 2 × 10^−10^ mbar. The system was equipped with a hemispherical electron energy analyzer (SPECS Phoibos 150), a delay-line detector and a monochromatic AlKα (1486.74 eV) X-ray source. High-resolution spectra were recorded at the normal emission take-off angle and with a pass-energy of 20 eV, which provided an overall instrumental peak broadening of 0.5 eV.

Attenuated total reflection Fourier transform infrared spectroscopy (ATR-FTIR) measurements of the GO, GO:CA (0:1) and the reduced samples were carried out using a Spectrum Two IR spectrophotometer (Perkin Elmer Inc., Waltham, MA, USA). All spectra were recorded in the wavenumber range between 400–4000 cm^−1^ at room temperature using a Deuterated-triglycine sulfate (DTGS) detector. Each ATR-FTIR spectrum is the average over 20 scans, using air as reference, and 2 cm^−1^ as the nominal spectral resolution.

Scanning electron microscopy (SEM) images were acquired on a SU-8230 SEM microscope (Hitachi High-Tech Corp., Tokyo, Japan) at 10 kV acceleration voltage. Several locations of each sample were scanned to ensure the representativeness of the micrographs.

High-resolution scanning transmission electron microscopy (HRSTEM) was performed in a HD-2700 STEM microscope (Hitachi High-Tech Corp., Tokyo, Japan) at 200 kV acceleration voltage equipped with an Energy Dispersive X-ray (EDX) Oxford detector (Oxford Instuments PLC, Oxford, UK). The samples were dispersed in ethanol using a probe-type ultrasonic homogenizer and deposited on standard Cu TEM grids with Formvar and Lacey Carbon polymeric films.

Thermogravimetric analysis (TGA) was conducted on a TGA-50 equipment (Shimadzu, Kyoto, Japan), under air flow, from room temperature to 700 °C and with a heating rate of 10 °C/min.

## 3. Results and Discussion

GO was synthesized by the improved Hummers method reported by Marcano et al. [9] and further reduced by an eco-friendly hydrothermal treatment in the presence of CA. The effect of CA in the reduction GO was studied using different GO:CA mass ratios. The starting and resultant materials were evaluated by different characterization techniques. The topographical and morphological properties were studied by SEM and scanning electron mode (STEM) techniques. The nanostructure was evaluated by HRSTEM, XRD, Raman and FTIR spectroscopies and the thermal stability was analyzed by TGA. The elemental quantification was determined by XPS.

### 3.1. Hydrothermal Reduction in the Presence of CA

**Figure 1:**
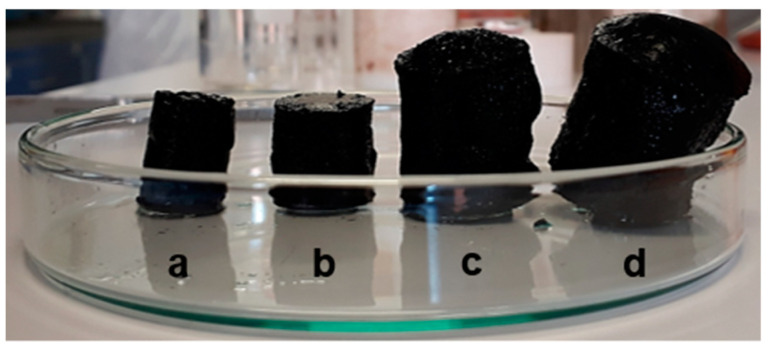
Optical image of the reduced graphene oxide (rGO) samples: (**a**) graphene oxide (GO):caffeic acid (CA) (1:0), (**b**) GO:CA (1:0.1), (**c**) GO:CA (1:0.5), and (**d**) GO:CA (1:1).

The optical images of the rGO materials obtained by the hydrothermal reduction of GO using the (1:0), (1:0.1), (1:0.5) and (1:1) GO:CA ratios are presented in Figure 1. The hydrothermal treatment of GO results in black monoliths. The effect of CA is observed by an increased volume of the resultant rGO materials. The self-assembly of the control sample GO:CA (1:0) occurs without templates or additional linkers, being only promoted by the hydrothermal conditions due to the π–π stacking and physical crosslinking of graphene sheets [29]. When the samples were reduced in the presence of CA, the self-assembly occurred due to π–π stacking between rGO sheets, and additionally between rGO sheets and CA molecules. The assembly of rGO into three-dimensional (3D) architectures due to π–π interactions is described in the literature [30] between natural phenolic acids (gallic acid, gentisic acid, protocatechuic acid, vanillic acid and ferulic acid) and GO upon heating. The increment of CA concentration leads to an increment of π–π interactions which is reflected into the increased monoliths’ volume observed in Figure 1, despite the same GO concentration used for the synthesis. The resultant rGO monoliths were dried and grounded to be characterized.

### 3.2. Characterization by SEM, STEM and HRSTEM

**Figure 2:**
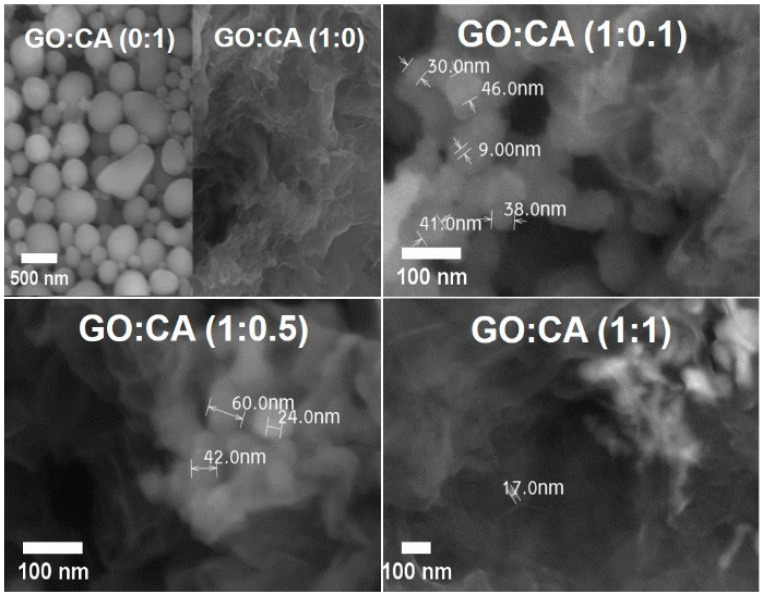
SEM micrographs of the reduced GO samples GO:CA (0:1), GO:CA (1:0), GO:CA (1:0.1), GO:CA (1:0.5) and GO:CA (1:1).

The microstructure of GO-CA samples was studied by SEM, as shown in Figure 2. The analysis of the control sample without GO, GO:CA (0:1), reveal the presence of spherical and non-spherical particles. The nanoparticles’ size, measured from the SEM micrographs, ranges between 5–300 nm. Agglomerates of particles are also observed. The quantitative analysis performed by energy dispersive X-Ray analysis (EDAX) in the secondary electron image of particles, revealed an average weight composition of 76% of carbon and 24% of oxygen. These nanoparticles result directly from the hydrothermal treatment of CA due to an apparent polymerization process. The polymerization of CA can be catalyzed by enzymes [31], UV light [32] or pyrolysis [33]. The UV-induced oxidative polymerization of CA requires the presence of oxygen and reactive oxygen species (ROS) [32]. The hydrothermal conditions and the oxygen present in the water used as a solvent should promote the CA polymerization. This simple hydrothermal synthesis can be of a great interest for future CA particle modifications.

The SEM micrographs of rGO materials, GO:CA (1:0), GO:CA (1:0.1), GO:CA (1:0.5), and GO:CA (1:1) are presented in Figure 2. These images display rGO agglomerates constituted by overlapped and folded rGO sheets. This morphology is in good agreement with the literature, being the typical morphology of rGO synthesized by hydrothermal treatment [24,29]. The removal of oxygen groups during the reduction turns the rGO sheets more hydrophobic, with the tendency to agglomerate to reduce the free energy. Moreover, the hydrogen bonding established between the remaining oxygen groups also contribute to the agglomeration of rGO sheets [34].

The samples reduced in the presence of CA, GO:CA (1:0.1), GO:CA (1:0.5), and GO:CA (1:1), present CA particles that can be easily distinguished among the rGO layers. These nanoparticles with a size only up to 60 nm are smaller than the particles observed in the GO:CA (0:1) control sample, which have sizes up to 300 nm. This fact may be explained due to space limitations inside the autoclave and due to the presence of rGO that prevents the nanoparticles from growing up freely.

**Figure 3:**
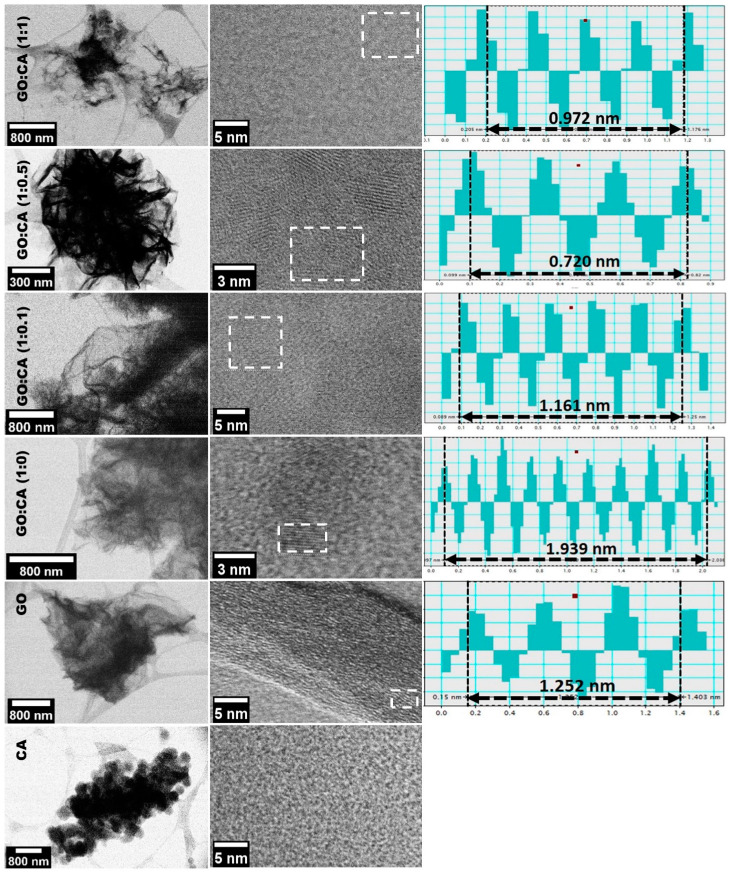
Scanning electron mode (STEM) (left column), high-resolution scanning transmission electron microscopy (HRSTEM) electron images (right column) and the respective determined profiles of hydrothermally reduced samples, GO and CA. The profile of CA sample could not be obtained as the sample was amorphous.

The morphological studies of GO:CA hybrids were complemented with the observation of the nanostructures using the scanning transmission electron microscope in both scanning electron mode (STEM) and high-resolution transmission electron mode (HRSTEM) as it can be seen in Figure 3. The STEM micrograph of CA presents an aggregate of particles with the edge transparent to electrons. The correspondent HRSTEM image reveals an amorphous structure, as shown in Figure 3. The STEM micrograph of the GO sample, in Figure 3, displays flat thin sheets of material, confirming the two-dimensional arrangement. The sheets seem to be overlapped as evidenced by the presence of lighter and darker regions. In the HRSTEM micrograph, it is possible to observe a certain degree of order perturbed potentially by the random distribution of oxygen-containing groups. In the micrographs of the reduced materials, displayed in Figure 3, it can be observed that the GO sheets became scrolled and folded after the reduction, which happens to turn the structure thermodynamically stable as previously reported in the literature [35]. The HRSTEM images of the reduced samples clearly reveal areas with very ordered graphitic layers, which supports the reduction of GO. Furthermore, the interplanar distance values were measured from the profiles which are determined in the white square marked areas in HRSTEM electron images. The interplanar distance values of rGO samples were found to be smaller than that of the GO sample, varying in the next arrangement: GO, 0.417 nm > GO:CA (1:0), 0.243 nm ≥ GO:CA (1:1), 0.243 nm > GO:CA (1:0.5), 0.240 nm > GO:CA (1:0.1), 0.232 nm, confirming the oxygen elimination through the reduction process. The measured *d*-spacings suggest that the crystallographic orientation visible in HRSTEM images, on the analyzed areas, is the (100) crystalline plane [36].

### 3.3. Structural Characterization by XRD, Raman and FTIR

**Figure 4:**
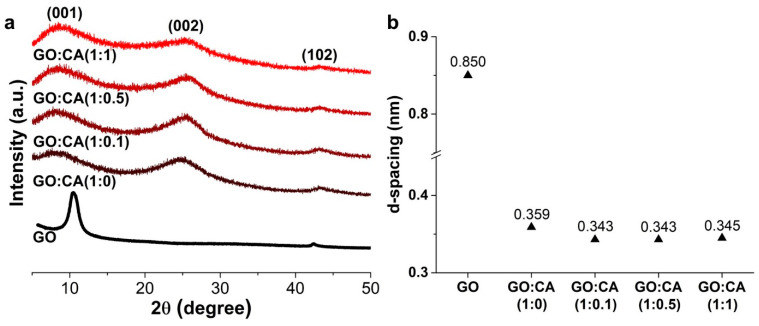
(**a**) XRD diffraction patterns and (**b**) the corresponding *d*-spacing value of GO and reduced samples: GO:CA (1:0); GO:CA (1:0.1); GO:CA (1:0.5) and GO:CA (1:1).

The XRD diffractograms of GO and rGO samples hydrothermally reduced with different amounts of CA are shown in Figure 4a. GO presents the characteristic (001) reflection at *d* ≅ 0.850 nm. The rGO samples also display the GO phase with the (001) reflection at *d* ≅ 0.883 nm. The appearance of the reflections (002) at *d* ≅ 0.356 nm and (100) at *d* ≅ 0.210 nm, typical of rGO, supports the efficiency of the hydrothermal reduction process in the presence of CA [37]. The distance between two GO or rGO layers is an important parameter to evaluate the amount of oxygen-containing functional groups between layers [38]. The *d*-spacings, Figure 4b, were calculated from the (001), (002) and (100) GO and rGO reflections, respectively. GO has a *d*-spacing of 0.850 nm, due to the high oxygen content resultant from the graphite oxidation. This value is higher than the one calculated in STEM, probably because of the distinct analyzed area. The reduced samples have lower *d*-spacing values than GO, considering the (002) orientation, varying in the following order: GO, 0.850 nm > GO:CA (1:0), 0.359 nm > GO:CA (1:1), 0.345 nm > GO:CA (1:0.5), 0.343 nm = GO:CA (1:0.1), 0.343 nm, confirming the oxygen removal during the reduction process. For the (100) orientation, the *d*-spacing has values of~0.200 nm. Both these *d*-spacings are consistent with the STEM analyses, which also show a decrease in the value of the *d*-spacing, although the only encountered orientation seems to be (100). The GO:CA (1:0) has the highest *d*-spacing among the reduced samples, as a consequence of the presence of more oxygen functionalities, which is an evidence of the role of CA in improving the GO deoxygenation. The GO:CA (1:0.1) and GO:CA (1:0.5) samples have the lowest *d*-spacing values, suggesting these CA amounts are optimal for the rGO reduction, which is also confirmed by HRSTEM analyses.

CA’s ability to reduce GO should be related to its high antioxidant activity [22,39]. In addition, it was reported that CA can polymerize when submitted to hydrothermal treatment, having improved oxygen scavenging activity [40]. The high antioxidant activity further enhanced by the potential polymerization of CA should be the explanation for the improved reduction of GO.

**Figure 5:**
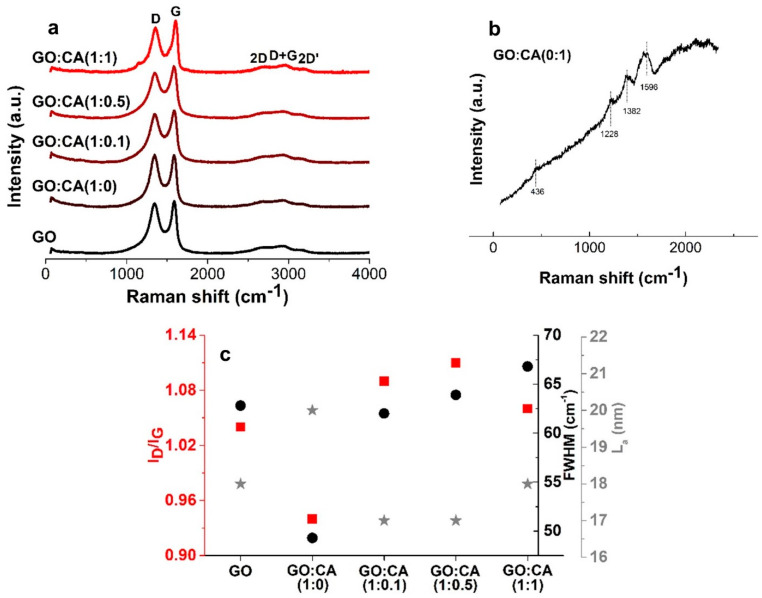
Raman spectra of (**a**) GO and reduced samples (**b**) GO:CA (0:1) sample. (**c**) D and G bands intensity (I_D_/I_G_) represented with red squares, full width at half maximum (FWHM) represented with black circles, and in-plane size of the sp^2^ domain (L_a)_ values represented with grey stars, of GO and reduced samples.

The Raman signature of graphitic materials characterized by the D, G, and 2D bands is present in all samples (Figure 5a). The D band appears between 1345–1356 cm^−1^ and represents the density of defects in the graphitic structure of the material. The G band, which appears around 1586–1606 cm^−1^, is a vibrational mode involving sp^2^ hybridized carbon atoms in graphene sheets [41]. The GO:CA (0:1) sample spectrum, shown in Figure 5b, presents bands at 439 cm^−1^ (o-diphenyl deformation, ring out-of-plane bending), 1228 cm^−1^ (C-O stretching), 1382 cm^−1^ (COO^−^ stretching) and 1596 cm^−1^ (C = C stretching) [42], that are not observed in the samples containing GO. The reduction of GO causes only a small variation of the ratio between the D and G bands intensity (I_D_/I_G_ ratio) Figure 5c, being also similar between rGO samples, which is in good agreement with the literature [25,43]. It should be noted that the I_D_/I_G_ ratios were calculated using the precise D and G bands’ intensities identified by performing spectra deconvolution involving a combined Gauss and Lorenz function.

The I_D_/I_G_ ratio is commonly used to estimate the in-plane size of the sp^2^ domains (*L*_a_) by applying Tuinstra–Koeing formula: $L_{a}=2.4\cdot 10^{-10}\cdot \lambda_{L\;}^{4}\cdot {\left(\frac{I_{D}}{I_{G}}\right)}^{-1}$ where $\lambda_{L}$ is the wavelength of the used excitation laser. One explanation for the increase in the I_D_/I_G_ ratio after reduction is the creation of new sp^2^ domains with smaller average sizes of about 17.6 nm, 17.3 and 18.1 nm for GO:CA (1:0.1), GO:CA (1:0.5) and GO:CA (1:1), respectively, shown in Figure 5c, than the pre-existing sp^2^ domains in the GO:CA (1:0) sample, which exhibited an average size of 20.5 nm [43]. It should be highlighted that the I_D_/I_G_ ratio value may be larger at the edges compared to the one exhibited at the basal plane, and also it can be influenced by many other factors [44]. So, the I_D_/I_G_ ratio could not be appropriated to show the impact of the reduction process. Instead, the full width at half maximum (FWHM) of the G band may be considered, as it does not vary significantly at the edges [45,46]. A continuous increase of the FWHM of the G band with the increase in CA amount was observed in the reduced samples, shown in Figure 5c, from 49.3 cm^−1^ for GO:CA (1:0) sample to 62.0 cm^−1^, 63.9 cm^−1^ and 66.8 cm^−1^ for GO:CA (1:0.1), GO:CA (1:0.5) and GO:CA (1:1) samples, respectively. This increasing trend clearly shows the impact of the presence of the CA on the reduction process of GO, which may be attributed to a continuous increase in the disorder level of the reduced samples.

The presence of CA residues between rGO sheets may also contribute to increase the disorder and consequently the I_D_/I_G_ ratio and FWHM_G_, which may explain the lowest values of these parameters observed for the GO:CA (1:0) sample. The samples with the highest I_D_/I_G_ and FWHM_G_ are GO:CA (1:0.1) and GO:CA (1:0.5). This observation may indicate that these samples were the most efficiently reduced, possessing the highest content of new graphitic domains [44]. The Raman results are in good agreement with the abovementioned HRSTEM and XRD *d*-spacing values, indicating again the materials GO:CA (1:0.1) and GO:CA (1:0.5) as the ones with the optimal GO:CA ratios for the GO reduction.

**Figure 6:**
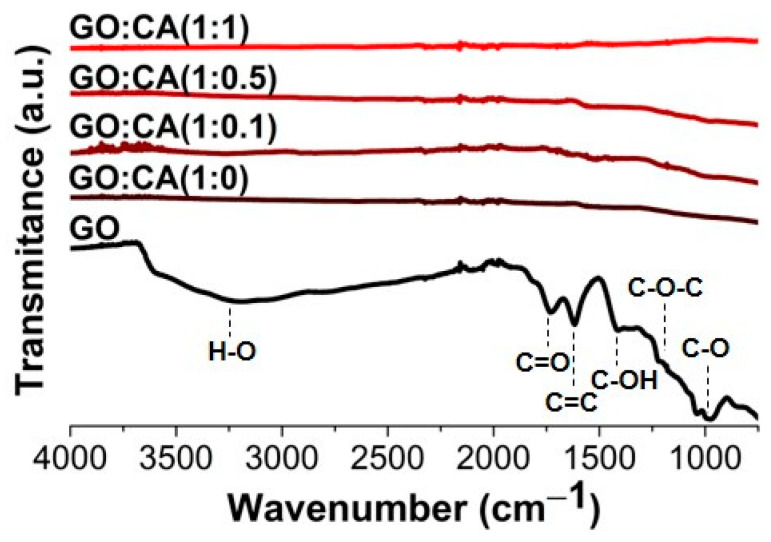
FTIR spectra of GO, GO:CA (0:1), and the samples reduced in the presence of caffeic acid GO:CA (1:1), GO:CA (1:0.5), GO:CA (1:0.1) and GO:CA (1:0).

FTIR spectroscopy was used to evaluate the presence of oxygen-containing groups in the structure of GO and in the reduced samples (Figure 6). The GO spectrum presents a broad band at 3192 cm^−1^, corresponding to the O-H stretching vibration, and peaks associated to the C = O (1734 cm^−1^), C-OH (1399 cm^−1^), C-O-C (1207 cm^−1^) and C-O (977 cm^−1^) vibrations. Similarly, the GO:CA (0:1) spectrum also presents oxygen-containing groups. After GO reduction with different amounts of CA, the oxygen-containing groups’ vibrations are not present in the FTIR spectra, which is an indication of the effective GO reduction [47,48].

### 3.4. Elemental Quantification by XPS

**Figure 7:**
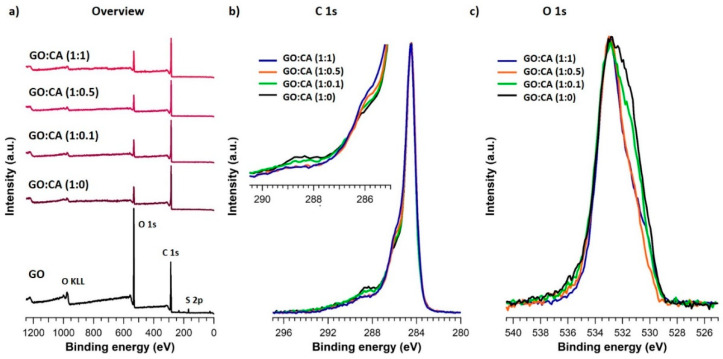
(**a**) XPS wide scans of hydrothermally reduced samples compared to GO. Normalized C 1s (**b**) and O 1s (**c**) high-resolution XPS spectra of the reduced samples. The upper inset of (**b**) shows a zoom-in of the key region of C 1s core level.

XPS was used to analyze the elemental composition as well as the chemical environment of the detected elements at a surface. Figure 7a shows the XPS wide scans obtained for GO (black spectra) and the reduced samples. While in the case of GO, the spectrum is dominated by the O 1s core level, the reduced samples showed its strong diminution with respect the C 1s. This behavior is described by the diminution of the atomic percentage of oxygen that declines from 37.6% in GO to values around 15% in the treated samples. Only very small differences were perceived between the reduced samples, being the order of oxidation degree GO:CA (1:1) > GO:CA (1:0) > GO:CA (1:0.5) > GO:CA (1:0.1) (Table S1, Supplementary Material). The sample GO:CA (1:1) has a C/O ratio of 4.78 that increases to 5.66 in GO:CA (1:0), 5.85 in GO:CA (1:0.5) and 5.99 in GO:CA (1:0.1).

C 1s high-resolution spectra resulted from all treated samples showed the typical shape of rGO (Figure 7b) [49]. Namely, a sharp peak related to the carbon network (284.5 eV) with two steps towards higher binding energies (BE), corresponding to different oxidation degrees of carbon, followed by a smooth tail due to π–π* electronic transfers. C 1s fitted spectra and atomic percentages of the components are shown in the Figure S1a and Table S1, Supplementary Material. The best fit was obtained by using five components: C1 for the carbon skeleton (C-C/C=C), C2 for C-O bonds, C3 related to C=O moieties, C4 for O-C=O and similar functional groups and C5 associated to the π–π* plasmon. C 1s peaks of the treated samples are similar but not equal. The inset showed in the upper part of Figure 7b details the differences noticed between the treated samples. The asymmetry near 286 eV, assigned to C-O bonds, enhances its intensity with an increasing concentration of CA in the reduction mixture. Conversely, the opposite effect was observed for the second shoulder (around 288 eV), associated to functional groups with a higher oxidation degree. Moreover, Figure 7c shows the comparison between the O 1s core levels obtained for the treated samples. The best fits were obtained by using three components (Figure S1b, Supplementary Material). The first one at around 531 eV associated with C = O bonds in aromatic compounds [49], the second one related to C-O type links [49], close to 533 eV, and a third small component due to water adsorbed, at approximately 535 eV (Figure S1b and Table S1, Supplementary Material). The comparison between the normalized O 1s spectra showed in Figure 7c indicates how the treated samples with a higher concentration of caffeic acid present lower intensity at the region related to oxygen in C=O bonds (about 531 eV) with respect to C-O.

The structural characterization of the GO:CA samples is resumed in Table 1. The hydrothermal treatment is an efficient method in reducing GO, which was confirmed by all the characterization techniques. The combination of the natural and non-toxic CA with the hydrothermal treatment promotes a higher reduction of GO when the CA is used in small amounts, especially using the GO:CA (1:0.1) ratio. Additionally, the use of CA during the hydrothermal treatment produces rGO decorated with CA nanoparticles. The CA nanoparticles resultant from the hydrothermal polymerization of CA are an advantage for functionalization. The rGO-CA materials are convenient to produce composite materials. The remnant oxygen groups derived from this mild reduction method, along with the CA nanoparticles, are useful to stablish chemical interactions with polymeric matrices. Moreover, the hydrothermal reduction in the presence of CA is an eco-friendly methodology. This green character represents an advantage over the typical chemical reduction using toxic reductants [14], or the high energy consumption thermal reduction under inert atmosphere [10].

### 3.5. Thermal Characterization by TGA

**Figure 8:**
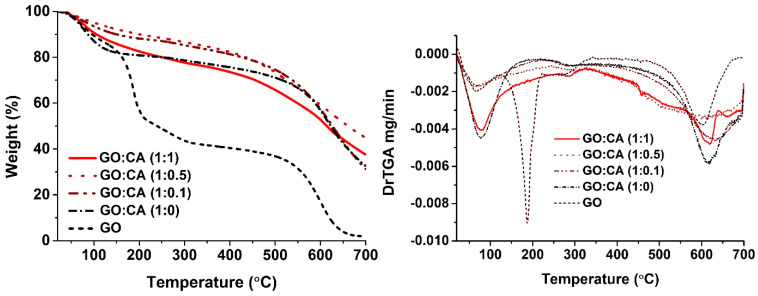
TGA and DTG curves of GO and rGO samples obtained under air atmosphere.

The thermal stability of GO and rGO was studied by TGA from 20 °C to 700 °C under air atmosphere, as can be seen in Figure 8. GO loses weight in three major steps. The first step happens below 120 °C and is attributed to the evaporation of water molecules entrapped in the GO structure. The second phase occurs approximately between 150–300 °C, corresponding to the elimination of oxygen functionalities. Finally, the last weight loss step starts approximately at 500 °C and refers to the degradation of unstable carbon [50,51]. The reduced samples follow a similar behavior, but with an improved thermal stability. Nevertheless, the rGO samples present mass losses during the first step, below 120 °C, corresponding to the elimination of adsorbed water and labile oxygen groups that were not fully removed during the reducing process. This result is expected from the mild reduction method described in this paper, since the extensive oxygen removal is only achieved using stronger reducing agents [50]. During the second degradation step, the reduced samples lose only less than 10% of weight, in opposition to a mass loss of 40% in the GO sample. The reduced samples were clearly more thermally stable than GO, confirming the elimination of oxygen functionalities during the hydrothermal reduction. The control sample GO:CA (1:0) presents a high degradation rate below 200 °C, meaning a high content of adsorbed water [52]. This result supports the improvement of the hydrothermal reduction by the combination with CA as a chemical reducing agent. The sample GO:CA (1:0.5) is the most stable among the samples reduced with CA, suggesting this ratio is ideal for the GO reduction.

## 4. Conclusions

This paper describes the preparation and characterization of rGO samples synthesized by a simultaneous combination of hydrothermal and chemical reduction processes. The effect of the GO:CA ratio in the hydrothermal reduction was investigated. The morphological characterization revealed the presence of CA particles, resultant from the hydrothermal CA polymerization. FTIR and XPS spectroscopy techniques confirm the reduction by the elimination/reduction of oxygen containing groups. Similarly, STEM and HRSTEM also support an efficient reduction with the presence of ordered graphitic layers. The XRD, Raman, XPS and TGA analyses confirm the improvement of GO hydrothermal reduction by the concomitant chemical reduction with CA. The samples GO:CA (1:0.1) and GO:CA (1:0.5) are suggested to be the optimal GO:CA ratios for the reduction process. The adoption of mild hydrothermal conditions and the use of a natural chemical reducing agent are efficient to reduce the GO. The hydrothermal treatment seems to be the major condition on the reduction of GO but the addition of a very small amount of CA maximizes the reaction and allows one to achieve a C/O ratio of 5.99, one of the highest reported if one considers just eco-friendly methods of rGO preparation [22]. The C/O ratio value achieved in this work (5.99) is 84% of the value reported by Bo et al., where the authors use about 50 times more CA. This green reduction strategy motivates the potential application of this rGO in biological, medical or food areas.

## Figures and Tables

**Table 1:** Resume of the GO–CA samples’ parameters measured by XRD, HRTEM, Raman and XPS characterization techniques.

	XRD	HRTEM	Raman			XPS
	*d*-Spacing (nm)	*d*-Spacing (nm)	I_D_/I_G_	FWHM	La (nm)	C/O
GO	0.850	0.417	1.04	62.8	18	1.66
GO:CA (1:0)	0.359	0.243	0.94	49.3	20	5.66
GO:CA (1:0.1)	0.343	0.243	1.09	62	17	5.99
GO:CA (1:0.5)	0.343	0.240	1.11	63.9	17	5.85
GO:CA (1:1)	0.345	0.232	1.06	66.8	18	4.78

## Supplementary Materials

The following are available online at https://www.mdpi.com/2079-4991/11/3/732/s1, Figure S1: (a) C 1s and (b) O 1s XPS spectra of GO and the samples hydrothermally reduced using different ratios of GO/caffeic acid. Best fit is also included. Table S1: Relative atomic percentages between carbon and oxygen in the diverse rGO samples (grey), and between the main components of C 1s (blue) and O 1s (green) XPS spectra.

## References

[1] Goli P., Ning H., Li X., Lu C.Y., Novoselov K.S., Balandin A.A. (2014). Thermal properties of graphene-copper-graphene heterogeneous films. Nano Lett..

[2] Wang J., Cao S., Sun P., Ding Y., Li Y., Ma F. (2016). Optical advantages of graphene on the boron nitride in visible and SW-NIR regions. RSC Adv..

[3] Zandiatashbar A., Lee G.H., An S.J., Lee S., Mathew N., Terrones M., Hayashi T., Picu C.R., Hone J., Koratkar N. (2014). Effect of defects on the intrinsic strength and stiffness of graphene. Nat. Commun..

[4] Vicarelli L., Heerema S.J., Dekker C., Zandbergen H.W. (2015). Controlling defects in graphene for optimizing the electrical properties of graphene nanodevices. ACS Nano.

[5] Zhang J., Zhang Z., Jiao Y., Yang H., Li Y., Zhang J., Gao P. (2019). The graphene/lanthanum oxide nanocomposites as electrode materials of supercapacitors. J. Power Sources.

[6] Liu Q., Shen J., Yang X., Zhang T., Tang H. (2018). 3D reduced graphene oxide aerogel-mediated Z-scheme photocatalytic system for highly efficient solar-driven water oxidation and removal of antibiotics. Appl. Catal. B Environ..

[7] Lee J.H., Choi H.K., Yang L., Chueng S.T.D., Choi J.W., Lee K.B. (2018). Nondestructive Real-Time Monitoring of Enhanced Stem Cell Differentiation Using a Graphene-Au Hybrid Nanoelectrode Array. Adv. Mater..

[8] Wang X.-Y., Narita A., Müllen K. (2018). Precision synthesis versus bulk-scale fabrication of graphenes. Nat. Rev. Chem..

[9] Marcano D.C., Kosynkin D.V., Berlin J.M., Sinitskii A., Sun Z., Slesarev A., Alemany L.B., Lu W., Tour J.M. (2010). Improved synthesis of graphene oxide. ACS Nano.

[10] Renteria J.D., Ramirez S., Malekpour H., Alonso B., Centeno A., Zurutuza A., Cocemasov A.I., Nika D.L., Balandin A.A. (2015). Strongly Anisotropic Thermal Conductivity of Free-Standing Reduced Graphene Oxide Films Annealed at High Temperature. Adv. Funct. Mater..

[11] Jha P.K., Singh S.K., Kumar V., Rana S., Kurungot S., Ballav N. (2017). High-Level Supercapacitive Performance of Chemically Reduced Graphene Oxide. Chem.

[12] Vázquez-Sánchez P., Rodríguez-Escudero M.A., Burgos F.J., Llorente I., Caballero-Calero O., González M.M., Fernández R., García-Alonso M.C. (2019). Synthesis of Cu/rGO composites by chemical and thermal reduction of graphene oxide. J. Alloys Compd..

[13] Tas M., Altin Y., Celik Bedeloglu A. (2019). Reduction of graphene oxide thin films using a stepwise thermal annealing assisted by L-ascorbic acid. Diam. Relat. Mater..

[14] Chua C.K., Pumera M. (2016). The reduction of graphene oxide with hydrazine: Elucidating its reductive capability based on a reaction-model approach. Chem. Commun..

[15] Mahmoud A.E.D. (2020). Eco-friendly reduction of graphene oxide via agricultural byproducts or aquatic macrophytes. Mater. Chem. Phys..

[16] Islam J., Chilkoor G., Jawaharraj K., Dhiman S.S., Sani R., Gadhamshetty V. (2020). Vitamin-C-enabled reduced graphene oxide chemistry for tuning biofilm phenotypes of methylotrophs on nickel electrodes in microbial fuel cells. Bioresour. Technol..

[17] Li J., Xiao G., Caibao C., Run L., Yan D. (2013). Superior dispersions of reduced graphene oxide synthesized by using gallic acid as a reductant and stabilizer. J. Mater. Chem. A.

[18] Miguel M., Barros L., Pereira C., Calhelha R.C., Garcia P.A., Castro M.Á., Santos-Buelga C., Ferreira I.C.F.R. (2016). Chemical characterization and bioactive properties of two aromatic plants: *Calendula officinalis L.* (flowers) and *Mentha cervina L.* (leaves). Food Funct..

[19] Ahmad N., Zuo Y., Lu X., Anwar F., Hameed S. (2016). Characterization of free and conjugated phenolic compounds in fruits of selected wild plants. Food Chem..

[20] Sato Y., Itagaki S., Kurokawa T., Ogura J., Kobayashi M., Hirano T., Sugawara M., Iseki K. (2011). In vitro and in vivo antioxidant properties of chlorogenic acid and caffeic acid. Int. J. Pharm..

[21] Mathew S., Abraham T.E., Zakaria Z.A. (2015). Reactivity of phenolic compounds towards free radicals under in vitro conditions. J. Food Sci. Technol..

[22] Bo Z., Shuai X., Mao S., Yang H., Qian J., Chen J., Yan J., Cen K. (2014). Green preparation of reduced graphene oxide for sensing and energy storage applications. Sci. Rep..

[23] Díez N., Śliwak A., Gryglewicz S., Grzyb B., Gryglewicz G. (2015). Enhanced reduction of graphene oxide by high-pressure hydrothermal treatment. RSC Adv..

[24] Mei X., Meng X., Wu F. (2015). Hydrothermal method for the production of reduced graphene oxide. Phys. E Low-Dimensional Syst. Nanostructures.

[25] Zhou Y., Bao Q., Tang L.A.L., Zhong Y., Loh K.P. (2009). Hydrothermal dehydration for the “green” reduction of exfoliated graphene oxide to graphene and demonstration of tunable optical limiting properties. Chem. Mater..

[26] Long D., Li W., Ling L., Miyawaki J., Mochida I., Yoon S.H. (2010). Preparation of nitrogen-doped graphene sheets by a combined chemical and hydrothermal reduction of graphene oxide. Langmuir.

[27] Chem J.M., Shen J., Yan B., Shi M., Ma H., Li N., Ye M. (2011). One step hydrothermal synthesis of TiO2-reduced graphene oxide sheets. J. Mater. Chem..

[28] Shen J., Shi M., Yan B., Ma H., Li N., Ye M. (2011). One-pot hydrothermal synthesis of Ag-reduced graphene oxide composite with ionic liquid. J. Mater. Chem..

[29] Xu Y., Sheng K., Li C., Shi G. (2010). Self-assembled graphene hydrogel via a one-step hydrothermal process. ACS Nano.

[30] Wang J., Shi Z., Fan J., Ge Y., Yin J., Hu G. (2012). Self-assembly of graphene into three-dimensional structures promoted by natural phenolic acids. J. Mater. Chem..

[31] Xu P., Uyama H., Whitten J.E., Kobayashi S., Kaplan D.L. (2005). Peroxidase-catalyzed in situ polymerization of surface orientated caffeic acid. J. Am. Chem. Soc..

[32] Behboodi-Sadabad F., Zhang H., Trouillet V., Welle A., Plumeré N., Levkin P.A. (2017). UV-Triggered Polymerization, Deposition, and Patterning of Plant Phenolic Compounds. Adv. Funct. Mater..

[33] Stadler R.H., Welti D.H., Stämpfli A.A., Fay L.B. (1996). Thermal decomposition of caffeic acid in model systems: Identification of novel tetraoxygenated phenylindan isomers and their stability in aqueous solution. J. Agric. Food Chem..

[34] Huang H.H., De Silva K.K.H., Kumara G.R.A., Yoshimura M. (2018). Structural Evolution of Hydrothermally Derived Reduced Graphene Oxide. Sci. Rep..

[35] Shen J., Hu Y., Shi M., Lu X., Qin C., Li C., Ye M. (2009). Fast and facile preparation of graphene oxide and reduced graphene oxide nanoplatelets. Chem. Mater..

[36] Matassa R., Orlanducci S., Tamburri E., Guglielmotti V., Sordi D., Terranova M.L., Passeri D., Rossi M. (2014). Characterization of carbon structures produced by graphene self-assembly. J. Appl. Crystallogr..

[37] Fathy M., Gomaa A., Taher F.A., El-Fass M.M., Kashyout A.E.H.B. (2016). Optimizing the preparation parameters of GO and rGO for large-scale production. J. Mater. Sci..

[38] Barra A., Ferreira N.M.N.M., Martins M.A.M.A., Lazar O., Pantazi A., Jderu A.A.A.A., Neumayer S.M.S.M., Rodriguez B.J.B.J., Enăchescu M., Ferreira P. (2019). Eco-friendly preparation of electrically conductive chitosan-reduced graphene oxide flexible bionanocomposites for food packaging and biological applications. Compos. Sci. Technol..

[39] Gülçin I. (2006). Antioxidant activity of caffeic acid (3,4-dihydroxycinnamic acid). Toxicology.

[40] Jin X.L., Yang R.T., Shang Y.J., Dai F., Qian Y.P., Cheng L.X., Zhou B., Liu Z.L. (2010). Oxidative coupling of cinnamic acid derivatives and their radical-scavenging activities. Chinese Sci. Bull..

[41] Wu J.-B., Lin M.-L., Cong X., Liu H.-N., Tan P.-H. (2018). Raman spectroscopy of graphene-based materials and its applications in related devices. Chem. Soc. Rev..

[42] Aguilar-Hernández I., Afseth N.K., López-Luke T., Contreras-Torres F.F., Wold J.P., Ornelas-Soto N. (2017). Surface enhanced Raman spectroscopy of phenolic antioxidants: A systematic evaluation of ferulic acid, p-coumaric acid, caffeic acid and sinapic acid. Vib. Spectrosc..

[43] Bai Y., Rakhi R.B., Chen W., Alshareef H.N. (2013). Effect of pH-induced chemical modification of hydrothermally reduced graphene oxide on supercapacitor performance. J. Power Sources.

[44] López V., Sundaram R.S., Gómez-Navarro C., Olea D., Burghard M., Gómez-Herrero J., Zamora F., Kern K. (2009). Chemical vapor deposition repair of graphene oxide: A route to highly conductive graphene monolayers. Adv. Mater..

[45] Claramunt S., Varea A., López-Díaz D., Velázquez M.M., Cornet A., Cirera A. (2015). The Importance of Interbands on the Interpretation of the Raman Spectrum of Graphene Oxide. J. Phys. Chem. C.

[46] Ghosh S., Ganesan K., Polaki S.R., Ilango S., Amirthapandian S., Dhara S., Kamruddin M., Tyagi A.K. (2015). Flipping growth orientation of nanographitic structures by plasma enhanced chemical vapor deposition. RSC Adv..

[47] Ossonon B.D., Bélanger D. (2017). Synthesis and characterization of sulfophenyl-functionalized reduced graphene oxide sheets. RSC Adv..

[48] Azizighannad S., Mitra S. (2018). Stepwise reduction of Graphene Oxide (GO) and its effects on chemical and colloidal properties. Sci. Rep..

[49] Hortigüela M.J., Machado D., Bdikin I., Neto V., Otero-Irurueta G. (2020). Chemical changes of graphene oxide thin films induced by thermal treatment under vacuum conditions. Coatings.

[50] Lavin-lopez M.P., Paton-carrero A., Sanchez-silva L., Valverde J.L., Romero A. (2017). Influence of the reduction strategy in the synthesis of reduced graphene oxide. Adv. Powder Technol..

[51] Sharma V., Jain Y., Kumari M., Gupta R., Sharma S.K., Sachdev K. (2017). Synthesis and Characterization of Graphene Oxide (GO) and Reduced Graphene Oxide (rGO) for Gas Sensing Application. Macromol. Symp..

[52] Chen H., Müller M.B., Gilmore K.J., Wallace G.G., Li D. (2008). Mechanically strong, electrically conductive, and biocompatible graphene paper. Adv. Mater..

