# Supplementary material for: Joining Caffeic Acid and Hydrothermal Treatment to Produce Environmentally Benign Highly Reduced Graphene Oxide
Source: Nanomaterials (Basel). 2021 Mar 15;11(3):732. doi: 10.3390/nano11030732 (PMC8001889; doi:10.3390/nano11030732)
Supplement: Supplementary file 1 [file nanomaterials-11-00732-s001.pdf]

Supporting Information

Joining Caffeic Acid and Hydrothermal Treatment to Produce Environmentally Benign Highly Reduced Graphene Oxide

Ana Barra [1,2,3], Oana Lazăr [4], Aida Pantazi [4,5], María J. Hortigüela [6], Gonzalo Otero-Irurueta [6], Marius Enăchescu [4,7], Eduardo Ruiz-Hitzky [3], Cláudia Nunes [2,*] and Paula Ferreira [1,*]

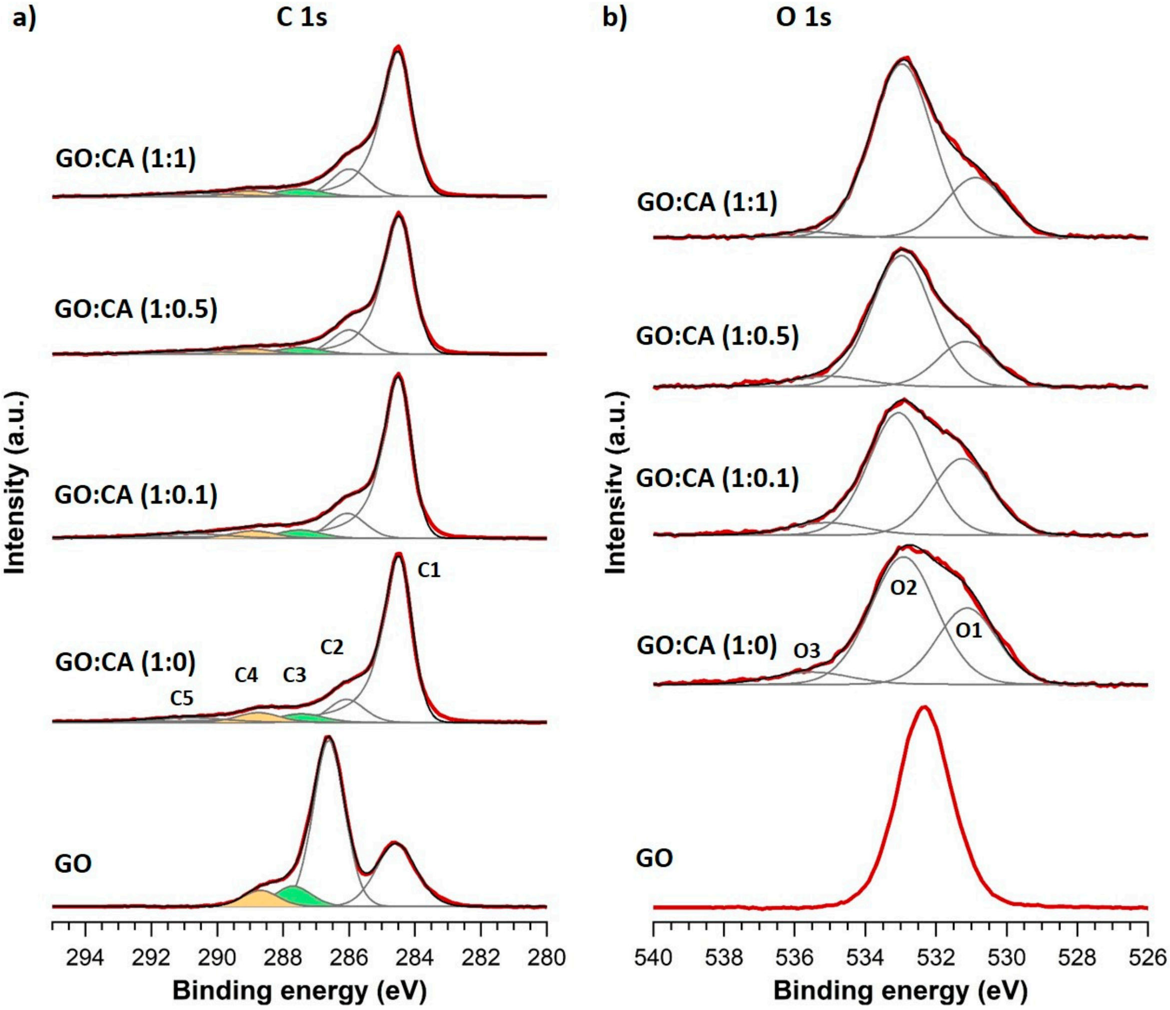

Figure S1. (**a**) C 1s and (**b**) O 1s XPS spectra of GO and the samples hydrothermally reduced using different ratios of GO/caffeic acid. In GO spectrum of the O 1s, the intensity was divided by five. Best fits are also included.

Table S1. Relative atomic percentages between carbon and oxygen in the diverse rGO samples (grey), and between the main components of C 1s (blue) and O 1s (green) XPS spectra.

Atomic %	BE (eV)	GO:CA (1:0)	GO:CA (1:0.1)	GO:CA (1:0.5)	GO:CA (1:1)	GO
C 1s		85	85.7	85.4	82.7	62.4
O 1s		15	14.3	14.6	17.3	37.6
C1 (C-C/C=C)	~ 284.5	68.9	68	69.5	68.9	27.8
C2 (C-O)	~ 286	13	14.8	16.0	17.1	57.6
C3 (C=O)	~ 287.5	6.2	6.1	5.6	5.8	8.3
C4 (O-C=O)	~ 289	6.6	5.6	4.7	3.9	6.3
C5 (π–π* transition)	~ 291	5.3	5.5	4.2	4.3	
O1 (O-C=O*/C=O aromatics)	~ 531	32.8	35.7	22.9	24.5	
O2 (C-O/O*-C=O)	~ 533	60.3	57.1	70.3	73.2	
O3 (water)	~ 535	6.9	7.2	6.8	2.3	
